# SEX DIFFERENCES IN THE ROLE OF SLEEP ON EXECUTIVE FUNCTION IN OLDER ADULTS

**DOI:** 10.1093/geroni/igad104.2955

**Published:** 2023-12-21

**Authors:** Yumiko Wiranto, Pilar Thangwaritorn, Kayla Meyer, Amber Watts

**Affiliations:** University of Kansas, Lawrence, Kansas, United States; University of Kansas, Lawrence, Kansas, United States; KU Alzheimer’s Disease Research Center, Fairway, Kansas, United States; University of Kansas, Lawrence, Kansas, United States

## Abstract

Advancing age is associated with a decline in sleep quality and cognitive performance. Previous work has shown that women report a higher number of sleep complaints, possibly due to hormonal and physiologic developmental changes across the lifespan. However, whether the effects of sleep quality on cognitive performance differ between sexes in older populations remains unclear. To address this gap, we analyzed data from 117 cognitively normal participants (44 men and 73 women) aged 60 and older (M = 74.82, SD = 6.52) to compare the effects of objectively measured sleep on executive function. Total sleep time was recorded with the GT9X Link Actigraph worn for seven consecutive days. The composite score of executive function was derived from a confirmatory factor analysis consisting of four separate tests (Verbal Fluency, Trail Making Test Part B, Digit Symbol Substitution Test, and Stroop Interference). We used hierarchical multiple regression to investigate sex differences in the effects of total sleep time on executive function. A quadratic model of total sleep time was a significant predictor of executive function in women (β = -.251, p = .034), but not in men adjusting for age and education. Longer sleep duration predicted improved executive function, but the performance decreased with oversleeping. These findings imply that interventions aimed at optimizing sleep duration may be beneficial for executive function in older women. Further research is necessary to identify sex-specific sleep requirements, which can inform the development of personalized interventions for older adults.

